# Supplementary material for: Influence of deficit irrigation levels on agronomic performance of pepper (Capsicum annuum L.) under drip at alage, central rift valley of Ethiopia
Source: PLoS One. 2023 Nov 22;18(11):e0280639. doi: 10.1371/journal.pone.0280639 (PMC10664897; doi:10.1371/journal.pone.0280639)
Supplement: Attachment — Submitted filename: review.docx [file pone.0280639.s001.docx]

1- English writing needs more work (Editing)
2- In the introduction section- the objective of the study is not clear need more elaboration
3- The results and discussion very acceptable and clear
4- The last section, Conclusion, the authors must think what is next and what should be the future work through presenting their recommendations.
