# Supplementary material for: Influence of deficit irrigation levels on agronomic performance of pepper (Capsicum annuum L.) under drip at alage, central rift valley of Ethiopia
Source: PLoS One. 2023 Nov 22;18(11):e0280639. doi: 10.1371/journal.pone.0280639 (PMC10664897; doi:10.1371/journal.pone.0280639)
Supplement: Attachment — Submitted filename: Response to Reviewers @pl.doc [file pone.0280639.s002.doc]

**Corresponding Author**

**Response to Academic Editor**

Dear Academic editor thanks very much for your valuable comments and suggestions.

1. I followed PLOS ONE style of writing a manuscript.
2. I stated funding information and financial disclosure statement in similar way.
3. I removed completely the reference “Ministry of Agriculture (MoA). Annual report on Irrigation development activities and its 766 constraints. Addis Ababa, Ethiopia. 2012, (unpublished document module 5).
4. In introduction part I added similar studies especially in physiological, biochemical and morphological changes due to deficit irrigation.
5. I tried to discussed and elaborated intensely the discussion and results parts.
6. Figure 1: Study area map image We decided to removed completely because it is not as such important.

**Response to reviewers**

Dear reviewers’ thanks very much for your valuable comments and suggestions.

**Reviewer 1**

1. As per your feedback I has been carefully editing the English syntax (grammar, spelling and fragments)
2. In introduction part I added similar studies especially in physiological, biochemical and morphological changes due to deficit irrigation, and rewrote the objectives
3. Even if the result and discussion are very acceptable and clear, I attempted to more elucidate on the occurrence/existence of implications in the experiment by linking to pertinent previous similar research and also tried to discussed deeply.
4. I clearly stated the conclusion and recommendation, and indicating the future work.

**Reviewer 2**

1. I improved English writing and editing (grammar, spelling and fragments).
2. In introduction part I added similar studies especially in physiological, biochemical and morphological changes due to deficit irrigation.
3. I tried to discussed deeply the discussion and results parts.
