# Supplementary material for: Influence of deficit irrigation levels on agronomic performance of pepper (Capsicum annuum L.) under drip at alage, central rift valley of Ethiopia
Source: PLoS One. 2023 Nov 22;18(11):e0280639. doi: 10.1371/journal.pone.0280639 (PMC10664897; doi:10.1371/journal.pone.0280639)
Supplement: Attachment — Submitted filename: Response to Reviewers @pl.doc [file pone.0280639.s003.doc]

**Corresponding Author**

**Response to Academic Editor**

Dear Academic editor thanks very much for your valuable comments and suggestions on revised manuscript.

1. As per your feedback of limited information on qualitative traits I have deeply seen the discussion part of the manuscript and improved the discussion on the result as much as possible by adding relevant informations. You can easily find the main corrections I already showed it in red color on the manuscript.
2. I followed PLOS ONE style of writing a manuscript and uploaded figure files to PACE so as to ensure figures meet PLOS requierements. As per the checked result all figures have met the PLOS requirements.

**Response to reviewer 2**

Dear reviewer thanks very much for your valuable comments and suggestions on revised manuscript.

1. According to your feedback of limited information on qualitative traits on discussion parts of the manuscript. I have profoundly seen the discussion on the results, and improved the discussion part as much as possible by adding and elaborating relevant informations. You find the main corrections easily; I already depicted it in red color on the manuscript.
